# Potential Modulation of Inflammation and Physical Function by Combined Probiotics, Omega-3 Supplementation and Vitamin D Supplementation in Overweight/Obese Patients with Chronic Low-Grade Inflammation: A Randomized, Placebo-Controlled Trial

**DOI:** 10.3390/ijms24108567

**Published:** 2023-05-10

**Authors:** Lena Kopp, Anna Schweinlin, Lina Tingö, Ashley N. Hutchinson, Viktoria Feit, Tabea Jähnichen, Katja Lehnert, Walter Vetter, Andreas Rings, Morten G. Jensen, Robert J. Brummer, Stephan C. Bischoff

**Affiliations:** 1 Department of Nutritional Medicine and Prevention, University of Hohenheim, 70599 Stuttgart, Germany; lena.stiefvatter@uni-hohenheim.de (L.K.); anna.schweinlin@uni-hohenheim.de (A.S.); viktoria.feit@med.uni-tuebingen.de (V.F.); t.jaehnichen@outlook.com (T.J.); andreas.rings@uni-hohenheim.de (A.R.); 2 Nutrition-Gut-Brain Interactions Research Centre, School of Medical Sciences, Örebro University, 70362 Örebro, Sweden; lina.tingo@oru.se (L.T.); ashley.hutchinson@oru.se (A.N.H.); robert.brummer@oru.se (R.J.B.); 3 Food and Health Programme, Örebro University, 70362 Örebro, Sweden; 4 Division of Inflammation and Infection, Department of Biomedical and Clinical Sciences, Linköping University, 72076 Tübingen, Germany; 5 Institute of Food Chemistry (170b), University of Hohenheim, 70593 Stuttgart, Germany; katja.lehnert@uni-hohenheim.de (K.L.); walter.vetter@uni-hohenheim.de (W.V.); 6 Haleon, 2665 Vallensbæk Strand, Denmark; mortengeorg.x.jensen@haleon.com

**Keywords:** obesity, overweight, chronic low-grade inflammation, probiotics, omega-3 fatty acids, hs-CRP

## Abstract

Obesity is characterized by low-grade inflammation and increased gut permeability. Here, we aim to evaluate the effect of a nutritional supplement on these parameters in subjects with overweight and obesity. A double-blinded, randomized clinical trial was conducted in 76 adults with overweight or obesity (BMI 28 to 40) and low-grade inflammation (high-sensitivity C-reactive protein (hs-CRP) between 2 and 10 mg/L). The intervention consisted of a daily intake of a multi-strain probiotic of *Lactobacillus* and *Bifidobacterium*, 640 mg of omega-3 fatty acids (n-3 FAs), and 200 IU of vitamin D (*n* = 37) or placebo (*n* = 39), administered for 8 weeks. hs-CRP levels did not change post-intervention, other than an unexpected slight increase observed in the treatment group. Interleukin (IL)-6 levels decreased in the treatment group (*p* = 0.018). The plasma fatty acid (FA) levels of the arachidonic acid (AA)/eicosapentaenoic acid (EPA) ratio and n-6/n-3 ratio (*p* < 0.001) decreased, and physical function and mobility improved in the treatment group (*p* = 0.006). The results suggest that hs-CRP may not be the most useful inflammatory marker, but probiotics, n-3 FAs, and vitamin D, as non-pharmaceutical supplements, may exert modest effects on inflammation, plasma FA levels, and physical function in patients with overweight and obesity and associated low-grade inflammation.

## 1. Introduction

### 1.1. Obesity and Low-Grade Inflammation

Obesity is a multifactorial disease reaching close to pandemic proportions [1,2]. Several diet-related diseases, such as cardiovascular disease, non-alcoholic fatty liver disease, and type 2 diabetes mellitus, are associated with obesity, and preventive methods are urgently needed [1]. Chronic low-grade inflammation seems to play a crucial role in the development of obesity-associated comorbidities such as insulin resistance, cardiovascular disease, and cancer [3]. Contrary to acute inflammation, defined as a rapid inflammatory response to an acute injury or infection, chronic systemic inflammation is characterized by a low-grade, continuous release of pro-inflammatory mediators [4]. The systemic inflammatory response in obesity is suggested to primarily originate from adipose tissue (AT), promoting infiltration of inflammatory cells (macrophages) and release of pro-inflammatory mediators, leading to systemic low-grade inflammation [5,6]. In support of this, previous studies have shown positive correlations between the volume of adipose tissue and the secretion of pro-inflammatory cytokines [5,7]. Another potential mediating factor is the altered gut microbiota in individuals with obesity [8], hypothetically leading to impaired intestinal barrier function and subsequent triggering of systemic inflammation [7,9]. In support of this, the gut microbial profile has been previously correlated with metabolic and inflammatory markers in individuals with obesity [10].

### 1.2. The Potential of Dietary Supplements

As outlined above, a potential strategy for preventing obesity-related ill-health may be to target the accompanying obesity-associated chronic low-grade inflammation. The use of non-pharmaceutical nutritional supplementation with omega-3 fatty acids (n-3 FAs), probiotic bacteria [11], and vitamin D has been previously investigated concerning other disorders with an inflammatory component [12]. The n-3 polyunsaturated fatty acids (PUFAs), especially 5,8,11,14,17-eicosapentaenoic acid (EPA) and 4,7,10,13,16,19-docosahexaenoic acid (DHA) that are substrates for the synthesis of lipid mediators (e.g., eicosanoids), have been shown to have anti-inflammatory effects [13]. Conversely, the omega-6 fatty acid (n-6 FA) arachidonic acid (AA; 20:4 n-6), which competes with EPA for enzymes, seems to be involved in pro-inflammatory processes [14]. Therefore, a balanced intake of n-6-to-n-3 FAs (n-6/n-3 ratio) is recommended [14,15], as higher n-6 intake has been previously associated with inflammatory diseases [16]. In support of this, previous studies investigating n-3 FA supplementation have shown an increase in anti-inflammatory eicosanoid and resolvins in individuals with obesity [17,18], and a reduction in pro-inflammatory cytokines, such as interleukin (IL)-6, IL-1β, and tumor necrosis factor (TNF)-α, in aging adults [19].

Furthermore, supplementing with probiotics could potentially alleviate inflammation by targeting the impaired gut barrier and altered gut microbiota. Several studies have investigated the impact of probiotics on inflammatory markers [20,21]. Strains of *Bifidobacterium* and *Lactobacillus* are associated with improvements in intestinal permeability and a reduction in inflammation [22,23,24]. In addition, n-3 FAs and vitamin D may have a positive impact on intestinal barrier function and microbiota composition [25,26], as well as inflammation [27,28,29]. However, there are few clinical studies investigating the dual supplementation of probiotics and n-3 FAs. A decrease in hs-CRP was found in people with overweight [30] and a study of the elderly showed modest effects on inflammation [31]. Additionally, supplementation with krill oil, probiotics, and vitamin D decreased inflammation in mice [32]. Dual supplementation of n-3 FAs and vitamin D in more severe cases of inflammatory disease may lack effects, as this showed no effect on hs-CRP in healthy and Crohn’s disease populations [33]. Here, we hypothesize that a dietary supplement consisting of probiotics, EPA + DHA, and vitamin D would help combat systemic inflammation in individuals with overweight and obesity by strengthening the intestinal barrier. A proof-of-concept study was designed to investigate the effect of eight weeks of daily supplementation with a multi-strain probiotic (two strains of *Lactobacillus* and two strains of *Bifidobacterium*), n-3 PUFAs, and vitamin D on inflammation (the primary outcome) and intestinal permeability (the secondary outcome) in subjects with overweight or obesity with chronic low-grade inflammation. The study also includes other secondary and explorative outcomes, such as fecal short-chain fatty acids (SCFAs) and physical function.

## 2. Results

### 2.1. Subject Characterization

**Figure 1:**
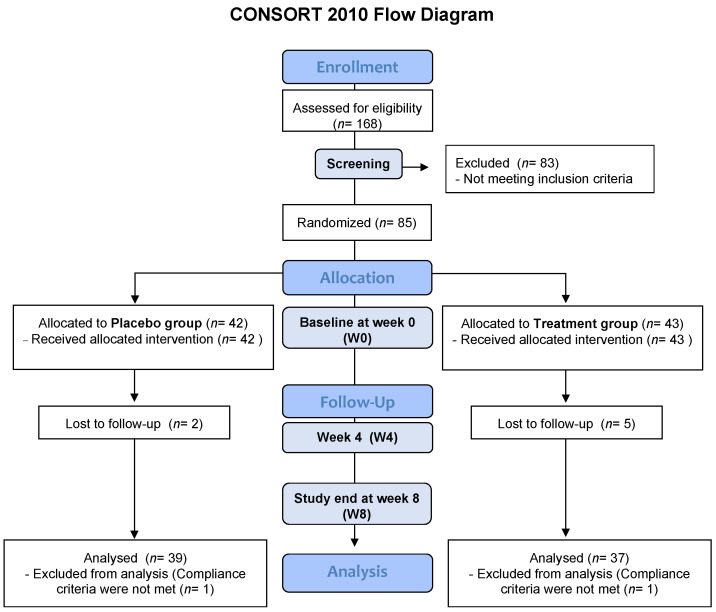
A study designed as a randomized, double-blind, placebo-controlled parallel-group study is shown as a flow diagram. A total of 85 participants were randomized into two groups at baseline (W0): 42 participants were in the placebo group and 43 participants were in the treatment group. The intervention took 8 weeks (W8) and the same parameters were measured at W0 and W8. Abbreviations: W, week.

The study was designed as a proof-of-concept, randomized, double-blind, 1:1 placebo-controlled parallel-group study with four visits (Figure 1). Recruitment took place between July 2019 and September 2020. After randomization and during the study, there were a total of seven dropouts due to antibiotic treatment, personal reasons, or loss of follow-up without any reason given. In addition, two participants did not meet the compliance criteria and were thus excluded from the analyses; 39 subjects from the placebo group and 37 subjects from the treatment group were finally analyzed (Figure 1). For more details, see Section 4.1 and Section 4.2.

Table 1 shows the participants’ baseline characteristics. Concerning body mass index (BMI), there were eight (20.5%) individuals with overweight (BMI 25–29.9 kg/m^2^) in the placebo group and none in the treatment group, while thirty-one participants (79.5%) were obese (BMI ≥ 30 kg/m^2^) in the placebo group and 37 (100%) in the treatment group (Table 1). There was, however, no statistically significant difference in BMI between the two groups. Notably, no laboratory parameter was significantly different at baseline between the two groups, including the primary outcome parameter, hs-CRP. Furthermore, the food frequency questionnaire (FFQ) showed no difference between the two groups; the results are listed in Table 1.

### 2.2. Effect of Supplementation on hs-CRP Levels and other Inflammatory Markers

**Figure 2:**
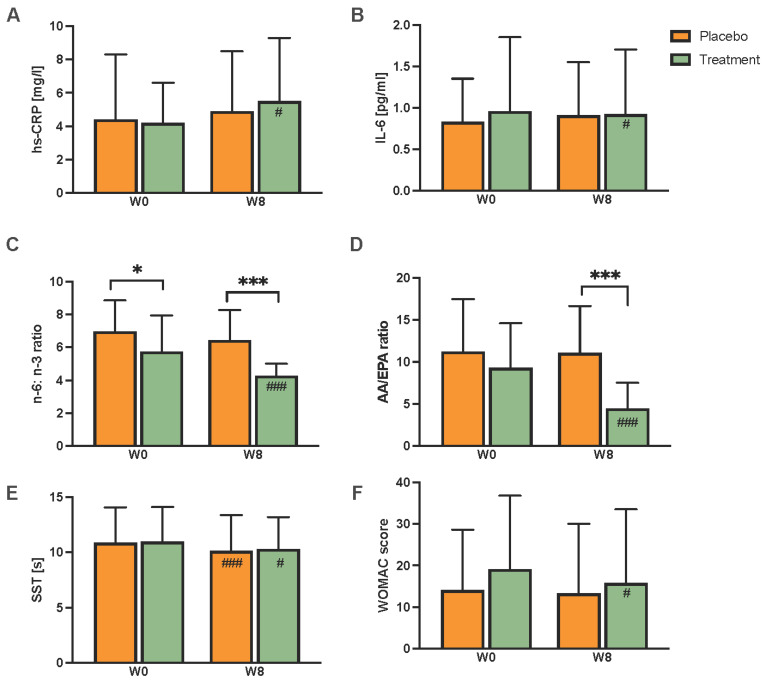
Effect of supplementation on hs-CRP levels (**A**) and IL-6 levels (**B**) comparing the placebo and treatment groups at study start (W0) and study end (W8). Further, FA plasma ratios were calculated as the n-6/n-3 ratio (**C**) and the AA/EPA ratio (**D**), as well as differences within and between groups at physical function tests Sit- and Stand test (**E**), and WOMAC score (**F**) before (W0) and after (W8) the eight-week intervention. Values are expressed as mean ± SD from 39 (placebo) and 37 (treatment) participants. Abbreviations: W, week; hs-CRP, high-sensitivity C-reactive protein, IL, interleukin; n, omega; AA, arachidonic acid; SST, Sit-to-Stand test; WOMAC, Western Ontario and McMaster Osteoarthritis Index. Statistics: # indicates differences between W0 and W8 within the treatment and placebo groups (calculated by either a paired *t*-test or Wilcoxon test). * Indicates differences between placebo and treatment group at W0 or W8. #, * *p* < 0.05; and ###, *** *p* < 0.001 (D-L, calculated by either unpaired *t*-test or Mann–Whitney U test).

Considering the primary endpoint hs-CRP, there was an increase from 4.2 ± 2.4 mg/L at W0 to 5.5 ± 3.8 mg/L at W8 in the treatment group (*p* = 0.018) and no changes in the placebo group (Figure 2A). Nevertheless, when comparing the two groups after treatment, there is no significant difference in mean hs-CRP levels or in treatment effects. The IL-6 level decreased within the treatment group from 1.0 ± 0.9 pg/mL at W0 to 0.9 ± 0.8 pg/mL at W8 (*p* = 0.034); however, neither a difference between groups nor a significant treatment effect was measured (Figure 2B). No other parameters of inflammation, such as TNF-α, interferon (IFN)-γ, and IL-4, -8, or -12, differed between the groups or were changed post-intervention (Table 2).

### 2.3. Laboratory Parameters

No change was observed between the groups for BMI, insulin, or the Homeostasis Model Assessment for Insulin Resistance (HOMA) index (Table 2). The glucose level increased within the placebo group (*p* = 0.041), while no changes were found within the treatment group (*p* = 0.650), nor between groups. Regarding the vitamin D levels, no significant change between groups was found; however, there was a modest increase of Δ0.51 ± 8.05 ng/mL in the treatment group and a slight decrease in the placebo group (Δ−0.33 ± 5.85 ng/mL) over the study period.

### 2.4. Fatty Acid Changes in Plasma

Regarding the ratio of inflammatory markers n-6 to n-3, a significant reduction (i.e., lower n-6 as compared to n-3 levels) was observed in the treatment group as compared to the placebo group (Figure 2C). The AA:EPA ratio was also reduced within the treatment group, from 9.3 ± 5.3 at W0 to 4.5 ± 3.0 at W8 (*p* < 0.001, Figure 2D). The ratio after the intervention at W8 was lower in the treatment group compared to the placebo group (*p* < 0.001). Regarding the total n-3 FAs, the treatment group showed higher values at W0 and W8 compared to the placebo group, as well as a significantly higher treatment effect compared to the placebo group. Additionally, the plasma n-6 FA level increased in both groups during the study (treatment group: *p* < 0.001; placebo group: *p* < 0.01) but no difference was measured in the treatment effect between both groups. Further FAs results are shown in Table S1.

### 2.5. Physical Function Tests

The Sit-to-Stand test (SST) time decreased in both groups, from 11.0 ± 3.1 s to 10.3 ± 2.9 s (*p* = 0.041) in the treatment group and from 10.9 ± 3.2 s to 10.1 ± 3.3 (*p* < 0.001) in the placebo group; no difference between the two groups was found (Figure 2E). The WOMAC score decreased from 19.1 ± 17.7 to 15.8 ± 7.7 in the treatment group (*p* = 0.006) and showed no changes in the placebo group. There was, however, no significant difference between the groups (Figure 2F and Table S2).

### 2.6. Gut Health

#### 2.6.1. Short-Chain Fatty Acids

The amounts of fecal SCFAs did not change over the study period, neither between the groups nor between different time points. However, a significant decrease in propionic acid was shown in the treatment group from 19.3 ± 8.7 μmol/g wet mass (20.4%) to 17.1 ± 9.6 μmol/g wet mass (18.3%) (*p* = 0.03; Table S2).

#### 2.6.2. Gut Barrier Markers

The multi-sugar urinary recovery test showed no changes within the groups or differences between the groups, and neither did the intestinal fatty acid-binding protein (I-FABP) and zonulin measurements (Table S2).

### 2.7. Correlation Analysis of All Data including All Participants

**Figure 3:**
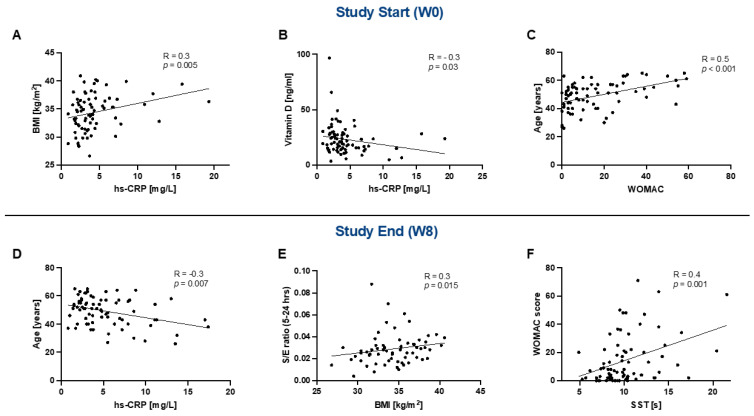
Correlations were conducted at study start (W0) (**A**–**C**) and showed a positive association between the BMI and hs-CRP (**A**), a negative association of hs-CRP with vitamin D (**B**), and a positive correlation between the WOMAC score and age (**C**). Correlations at study end (W8) (**D**–**F**) showed a negative correlation between hs-CRP and age (**D**), a positive association between the BMI and the sucralose/erythritol ratio (**E**), and a positive correlation between the SST and WOMAC score (**F**). Abbreviations: BMI, body mass index; hs-CRP, high-sensitivity C-reactive protein; WOMAC, Western Ontario and McMaster Osteoarthritis Index. SST, Sit-to-Stand test; S/E ratio, sucralose/erythritol ratio. Statistics: Correlations are shown as scatterplots with Spearman’s rho as the correlation coefficient.

Correlation analyses were conducted separately for W0 and W8. Baseline (W0) correlation analysis showed a positive association between BMI and hs-CRP, and a negative correlation between vitamin D level and hs-CRP (Figure 3A,B). Additionally, a positive correlation was seen between a higher age and the WOMAC score; a higher score is associated with higher self-reported physical disability status (Figure 3C). Correlation analysis between parameters measured at W8 showed a negative correlation between hs-CRP and the age of the study participants (Figure 3D), while a positive correlation was shown between the sucralose/erythritol ratio and the participant´s BMI (Figure 3E). A positive correlation between the WOMAC score and the SST was also found (Figure 3F).

### 2.8. Subgroup Analysis

**Figure 4:**
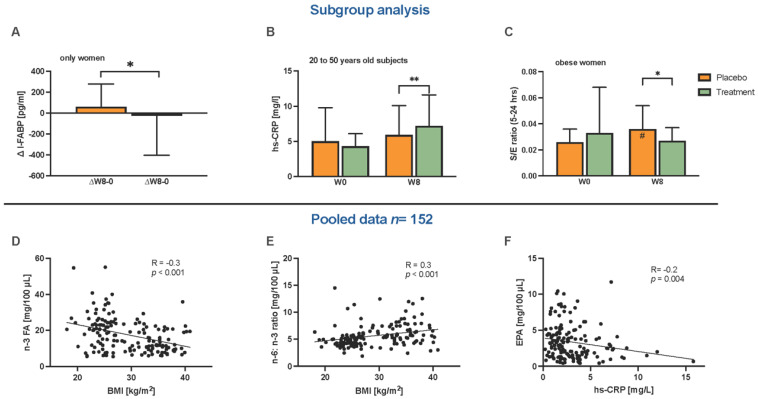
Subgroup analysis (**A**–**D**) and correlation analysis of pooled data from the parallel group with the elderly (**D**–**F**) were conducted. The subgroup analysis with only women showed a positive reduction of the ΔI-FABP within the treatment group and a difference between the treatment and placebo groups (placebo n = 30, treatment n = 30) (**A**). Only women with obesity showed a difference at the W8 time point of the S/E ratios (5–24 h) between the placebo and treatment groups (placebo n = 21, treatment n = 27) (**B**). Regarding the age categories of the 20–50 year old subjects, the hs-CRP level at W8 was higher in the treatment group compared to the placebo (placebo n = 20, treatment n = 16) (**C**). By pooling the present cohort and aged participants from a partner study (n = 152) at the W8 time point, Spearman’s-rank correlation (R) showed a negative association between n-3 PUFA levels and the BMI (**D**), a positive correlation of the n-6/n-3 ratio in plasma to the BMI (**E**), and a negative correlation between EPA plasma levels and hs-CRP (**F**). Abbreviations: BMI, body mass index; EPA, eicosapentaenoic acid; hs-CRP, high-sensitivity C-reactive protein; n-3 PUFA, omega-3 polyunsaturated fatty acids; n-6/n-3 ratio, omega-6-to-omega-3 ratio. Statistics: Grouped bar plots (**A**–**C**) and correlations are shown as scatterplots with Spearman’s rho as correlation coefficient (**D**–**F**). # indicates differences between W0 and W8 within the group. * Indicates differences between the placebo and treatment groups at W0 or W8. #, * *p* < 0.05; ** *p* < 0.01.

We also conducted a set of subgroup analyses in the current study. When only analyzing women, ΔI-FABP (W8-W0) was negative, indicating a reduction over the study period within the treatment group (−22.04 ± −379.00 pg/mL), while with the females in the placebo group, the delta was positive (61.89 ± −217.58 pg/mL); rendering the ΔI-FABP significantly lower in the treatment group as compared to the placebo group (*p* = 0.044; Figure 4A) when comparing only women. Dividing the participants into low and high age groups (between 20 and 50 years, and between 51 and 65 years, respectively) showed that the hs-CRP level at W8 was higher among the younger subjects within the treatment group as compared to the placebo group (*p* = 0.004, Figure 4B). In another subgroup analysis including only women with obesity, the S/E ratio (5–24 h) increased within the placebo group over the study period (*p* = 0.030; Figure 4C). Comparing the placebo and treatment groups, the S/E ratio at W8 was lower in the treatment group compared to the placebo (*p* = 0.034, Figure 4C). Moreover, by pooling the participants from our study with the elderly subjects from the Örebro cohort (i.e., 79 elderly and 73 overweight people, data for the elderly are already published [32]), three correlations arose: a negative correlation between n-3 PUFA in plasma and BMI, as a higher BMI is associated with a lower n-3 PUFA plasma concentration (Figure 4D); a positive correlation between the n-6/n-3 ratio in plasma and BMI, as a higher BMI is associated with a higher ratio (Figure 4E); and a negative correlation between EPA in plasma and hs-CRP levels, as a higher EPA level is associated with a lower hs-CRP level (Figure 4F).

## 3. Discussion

The present study aimed to investigate the effect of an eight-week daily supplementation with probiotics (*Lactobacilli* and *Bifidobacteria* strains), EPA + DHA, and vitamin D on low-grade inflammation in individuals with overweight and obesity. The study showed no difference in the primary outcome parameter, hs-CRP levels. However, IL-6 levels decreased in the treated subjects’ post-intervention, which may indicate some modest effects on inflammation. In addition, the intervention seemed to elicit some improvements in mobility, as evaluated by self-reported WOMAC scores, in the treatment group. Regarding the measurements of gut barrier function, we found no differences between the intervention and the placebo group, other than some findings relating to sex and age in our subgroup analyses.

This study sprung out of the idea that a hypothetical remedy for obesity-related ill-health is lowered systemic inflammation. Yet, contrary to our expectations, the treatment did not decrease hs-CRP, but rather led to a slight increase. These findings also seem to conflict with most previous results from similar studies described in the literature. For example, a recent meta-analysis suggests a significant small to medium reduction of CRP after probiotics (−0.43 mg/L) and n-3 FA (−0.17 mg/L) supplementation in middle-aged and older adults [34]. Dual supplementation combining probiotics with n-3 PUFAs and vitamin D, as tested here, has only been investigated once before (except the parallel study we performed in Örebro, Sweden) by Rajkumar et al., showing a small reduction in hs-CRP levels from 5.60 to 5.26 mg/L in the treated subjects [30]. Comparing that study with the current one, there are major differences in the demographics of the participants. In our treatment group, the mean BMI was 35.0 kg/m^2^ as compared to 28.79 kg/m^2^ in the study by Rajkumar et al. Since higher BMI is associated with increased CRP levels [35,36], which was also confirmed in our baseline correlations, there is a possibility that the supplementation was not sufficient to address the more severe inflammatory status in our subjects with obesity. Another reason could be the age difference, as we also included younger participants (26–65 years of age), whereas the study by Rajkumar et al. recruited 40–60-year-olds [30]. Interestingly, our correlation analysis showed that hs-CRP was lower with increasing age, which was also confirmed in our subgroup analyses. Hence, the younger subjects seem to contribute more to increasing hs-CRP in our study. This could have multiple causes; for example, hormone therapies and the menstrual cycle may alter hs-CRP levels [37,38,39], which may be a factor to consider as many women participated (~79%) in this study. Such medications were not recorded in our study, but as we included women of reproductive age, and hence this factor may have a potential influence on our hs-CRP measurements.

Other factors that may have some bearing on the lack of significant hs-CRP decreases in the current study may be the ineffectiveness of the supplement due to dosage or the inclusion of probiotic strains. In comparison, Rajkumar et al. used a higher dosage and more bacterial strains, such as *Streptococcus salivarius* subsp. *Thermophilus*, and some additional strains of *Bifidobacteria* and *Lactobacilli* [30]. Besides the bacterial strains and the dosage of probiotics, another factor that may play a role is the dosage of EPA and DHA. Although the dose of fish oil administered here is equivalent to the recommended daily intake for healthy adults [40], other studies showed that a higher dosage of EPA and DHA intake (of more than 2 g per day) seemed to be more successful in achieving anti-inflammatory effects [41]. The negative correlation between plasma EPA and hs-CRP found in this study potentially gives some support in this direction; it was also previously confirmed in healthy subjects [42]. Furthermore, alluding to a potentially insufficient dosage, the vitamin D dose may also have benefited from some adjustments, as a previous study showed that a dose of 1000 IU/d resulted in a decrease in hs-CRP levels [43]. The current dose may perhaps be considered a preventive dose but may not be high enough to elicit therapeutic effects.

An additional factor of failure in hs-CRP reduction was the within-person variability from screening to the study start. These findings are consistent with the parallel study with the elderly [31] and other studies [44,45]. Various factors, such as age, gender, smoking, lipid levels, blood pressure, infections, and other environmental exposures, may contribute to individual variations in hs-CRP levels [46,47]. On the basis of its high intra-individual variations, it is possible that hs-CRP is not the most useful inflammatory marker in studies adopting this design. Perhaps more screening and follow-up samples are needed, i.e., repeated hs-CRP measurements, to ensure elevated levels over time. Or other inflammatory markers, for example, IL-6, may be better suited.

Regarding IL-6 and the other evaluated inflammatory markers, the current study showed a potential health benefit by reducing IL-6 levels within the treatment group, albeit modestly, Δ−0.04 ± 0.62. Circulating IL-6 is elevated in individuals with obesity [48], and AT is one of the main sources of this inflammatory mediator [49]. Healthy individuals have low average levels of less than 3 pg/mL to 4 pg/mL, while levels can be as high as 9 pg/mL in individuals with obesity [49]. As the participants in the current study had levels between 0.8–1 pg/mL, no particularly severe inflammation seemed to be present as assessed by IL-6. Nevertheless, the decrease in IL-6 may suggest a slight improvement in inflammation in the treatment group. These effects are consistent with a systematic meta-analysis, showing that probiotics (Δ−0.68 pg/mL) and n-3 FA (Δ−0.19 pg/mL) have a significant effect on lowering IL-6 levels [34]. Anti-inflammatory effects have also been previously shown for our selected probiotic strains, e.g., *Lactobacillus paracasei* improved the inflammatory status in mice with type 2 diabetes [23]. Neither of the other measured inflammatory markers changed significantly throughout the study.

Moreover, other indicators may suggest some benefits from the combined supplementation in this trial. For example, in improving the FA composition in blood and tissues. The Western diet is characterized by increased n-6 intake and therefore a higher n-6/n-3 ratio, which could be as high as 15:1 or 16:1 [50]. Supplementation with n-3 FA or a balanced n-6/n-3 diet can improve the balance [51]. One possible hypothesis is that overweight individuals with a Western diet often have a less favourable ratio of these FA, which promotes a pro-inflammatory state [50]. Our correlation analysis confirmed this, as a higher BMI was associated with a higher ratio of n-6 and n-3 FAs, and a higher BMI was associated with a lower intake of n-3 FAs. One study already showed that a reduced intake of n-6 FA can improve obesity-related disorders [52]. Therefore, improving this ratio could have health benefits. In the current study, the treatment group showed an improvement in the n-6/n-3 ratio from 6:1 to 4:1. This ratio is similar to that of the Japanese population [53], whose lifestyle is associated with health [54]. Other previous studies have shown that ratios of 4:1 are associated with a reduction in mortality from heart disease [55], and a ratio of 1:1 to 3:1 suppresses inflammation in patients with rheumatoid arthritis [50] and prevents the risk of other chronic diseases [16]. Therefore, the result showed a potential anti-inflammatory effect. This could be confirmed by a reduced AA:EPA ratio, which diminished from 9.3 to 4.4 after eight weeks of supplementation within the treatment group. An unbalanced AA:EPA ratio is associated with the development of obesity [56] and cardiovascular disease [57]. Higher AA:EPA ratios tend to induce inflammation, while lower ratios improve arterial stiffness in patients with obesity and dyslipidemia [58]. Thus, it seems our treatment group reached the range of a preventive ratio, while the placebo group of this study remains in the pro-inflammatory range with an AA/EPA ratio of 11. With a longer intervention and follow-up period, the anti-inflammatory effects attributable to these changes in PUFA ratios might have occurred. A meta-analysis, for example, shows that a longer duration of intake could lead to a stronger IL-6-lowering effect [59]. On the basis of our results, we cannot comment further on any influence of vitamin D supplementation on inflammatory markers, except that the correlation analysis showed a negative association between vitamin D level and measured hs-CRP levels at baseline, which could indicate an anti-inflammatory effect. The above-mentioned meta-analysis could not show any association between vitamin D and significant reductions in inflammatory biomarkers [34]. In summary, the effect of probiotics, n-3 FA, and vitamin D on low-grade inflammation in individuals with obesity needs further investigation as this field, considering the current study, shows inconclusive results. On this note, dietary modification in general and weight loss could further alter inflammatory markers, as shown in other studies [60,61].

Regarding mobility, obesity is usually associated with reduced mobility. Therefore, an improvement in mobility markers is beneficial. The study showed an improvement in the WOMAC score in the treatment group. This improvement could possibly indicate a subtle anti-inflammatory effect leading to a reduction in pain during certain movements. Results from other studies have shown similar effects after supplementation with n-3 PUFAs [62], probiotics [63], and vitamin D [64]. Our subgroup correlation analysis showed a positive association between age and WOMAC score, which worsens with age, as expected. This association may suggest that the pain-reducing effect is more important in older people.

However, many overweight people of all ages have limited mobility. The results of the SST suggest an improvement in mobility after the intervention; however, this is in both groups, which could be due to a ‘learning effect’. Overall, further mobility markers should be investigated in future studies. Although the results have modest effects, an improvement in mobility markers in this population is a particularly valuable effect. An anti-inflammatory effect may be associated with more exercise and, therefore, possible weight loss, leading to less stress on the knees and hips [65]. Supplementation is, thus, a good start to reducing pain and inflammation, which, in turn, leads to better mobility.

In terms of gut health benefits, we expected an improvement in intestinal permeability, as n-3 PUFAs, probiotics, and vitamin D have been previously suggested to positively impact intestinal barrier function [11,26,66]. Here, we detected no improvements in gut permeability (measured by the sugar test, I-FABP, or zonulin). The correlation analysis, however, confirms a previously found positive association between intestinal permeability (by sucralose/erythritol (S/E) ratio) and BMI [67]. Additionally, our subgroup analyses showed some minor changes. For example, women with obesity had a lower S/E ratio after the intervention as compared to placebo, and the women-only group showed an improvement in I-FABP. These observations suggest that the supplementation may have some subtle effects on colon permeability that are more pronounced in females. Furthermore, since we supplemented with probiotics, a change in the composition of the microbiota was expected. We hypothesized that there would be an increase in SCFA, especially since the probiotic strain *Bifidobacterium* spp. is known to (mainly) produce acetic acid, and *Lactobacillus acidophilus* produces propionic acid, butyric acid, and acetic acid [68]. The supplementation did, however, not lead to any major changes in SCFA; a reduction in propionic acid in the treatment group occurred, but this does not show any associations with any of the evaluated inflammatory markers. However, not only probiotics, but also n-3 FAs may have effects on an imbalanced microbiota in obesity by increasing SCFA-producing bacteria [69]. Nevertheless, it seems that the actual probiotic and EPA and DHA dosing had no sufficient effect on SCFA production in this study on overweight and obese individuals.

To assess compliance, which is yet another important factor to consider in terms of the effectiveness of an intervention, plasma FA and vitamin D levels were measured. The increase in participants’ EPA and DHA levels, as well as n-3 and n-6 levels, confirmed the bioavailability of the supplement and participant compliance. Counterintuitively, the participants in the placebo group also showed increased n-3 levels post-intervention. This could be because all subjects received the same information about the intervention and the benefits of n-3 FAs, which may have led to an unintended change in their diet. The results show, however, that a continuous intake of the n-3 capsules leads to a significant reduction of the n-6/n-3 ratio in the treatment group, and therefore future health benefits, after prolonged intake, could potentially be expected. Regarding the vitamin D level, the general serum level before and after the supplementation was very low; levels greater than 32 ng/mL are recommended [70]. Contrary to expectations, vitamin D supplementation did not lead to an increase in blood levels. It is possible that the dosage in our study was not sufficient. There may also be other factors hindering vitamin D-driven effects; middle Europe has relatively fewer hours of sunlight compared to many other parts of the world, and vitamin D levels tend to be low in the general population, especially during the fall and winter seasons [71]. This could, in part, explain the lack of increase in plasma within the treatment group.

## 4. Materials and Methods

### 4.1. Participant Selection

Female and male subjects with overweight (body mass index (BMI) 28–29.9 kg/m^2^) and obesity (BMI ≥ 30–40 kg/m^2^) aged between 25 and 65 years were screened for low-grade inflammation (serum hs-CRP level between 2 and 10 mg/L). Participants had to be free from chronic illness (except for obesity-associated diseases like hypertension, non-alcoholic fatty liver disease, and depression) and willing to adhere to certain dietary rules (i.e., no significant changes in diet, fiber intake, or fluid intake throughout the study period, and no intake of probiotic products, n-3 PUFA supplements, or vitamin D supplements, in addition to adhering to all instructions concerning the study procedures). Additionally, habitual physical activity levels should be maintained throughout the study. The following exclusion criteria were also adopted: any previously diagnosed gastrointestinal disease, type 1 or 2 diabetes mellitus, antibiotic or steroid medication within the last four months, intake of probiotics within the last four weeks, frequent use of non-steroidal anti-inflammatory drugs, intake of any other drugs affecting inflammation and the gastrointestinal barrier, rigorous physical exercise for more than four hours/week, known allergies to fish, soy or milk, regular smoking, alcohol consumption of more than 108 g pure alcohol per week or more than 36 g pure alcohol per occasion, as well as pregnancy and breastfeeding. The inclusion and exclusion criteria listed above remained unchanged throughout the study. The study was conducted according to the Declaration of Helsinki at the Center for Clinical Nutrition Stuttgart in 2019 and 2020 and has been approved by the local Ethical Committee (Ethik-Kommission der Landesärztekammer Baden-Württemberg, F-2018-105), and was registered at ClinicalTrials.gov (NCT04126330).

### 4.2. Study Design, Visits, Power Calculation and Outcome Parameters

#### 4.2.1. Study Design

The study was designed as a proof-of-concept, randomized, double-blind, 1:1 placebo-controlled parallel-group study with four visits (Figure 1). The primary outcome parameter was the post-interventional effect on hs-CRP levels within and between groups.

#### 4.2.2. Power Calculations

The study was powered to show a 30% improvement (i.e., lowering) of hs-CRP in the treatment group post-intervention. These estimations were based on previous data generated by our research group in the same study population (unpublished consecutive data of an in-house weight reduction program); a standard deviation of 0.61 for log-level hs-CRP was expected. After accounting for a maximum dropout rate of 20% and the power to detect statistical significance at the level of 5%, we estimated that we could include 90 subjects, i.e., 45 participants in each arm.

#### 4.2.3. Additional Outcome Parameters

Secondary outcome parameters were other inflammatory markers (i.e., IL-4, 6, 8, 10, and 12 TNF-α), intestinal barrier function (as measured by a multi-sugar urine recovery test), physical function (evaluated by the Western Ontario and McMaster Universities Osteoarthritis Index (WOMAC) questionnaire and the STS), fasting blood glucose and insulin levels, n-6/n-3 ratio in plasma, and I-FABP. Exploratory outcome parameters included 25-hydroxy (OH)-vitamin D level in plasma, the intestinal barrier marker zonulin, and SCFA analysis in feces. The FFQ was filled out at the bassline for a descriptive argument.

#### 4.2.4. Study Visits

Please refer to Figure 1 for a schematic overview. At the baseline visit (W0, Screening) the participants received information about the study and signed an informed consent form, the BMI was calculated, and blood samples (approximately 4 mL) were obtained for hs-CRP measurement. If the participants fulfilled all inclusion (and no exclusion criteria) they were subsequently invited to a second visit (i.e., baseline/W0) where all baseline parameters were assessed. Before attending this visit, all included participants had picked up the instructions and material for the fecal sample collection and the urine recovery test, all of which were collected in their home before attending W0. Four weeks after W0 they came back for the third visit (W4) and after another four weeks (eight weeks after W0) they completed their final visit (W8). All study products were distributed at W0, but before the supplementation commenced, fasting blood samples were taken to analyse hs-CRP, other blood markers, and FA. Additionally, the fecal samples and urine collected at home (subsequently used for SCFA and the barrier permeability measurements) were handed in to the research team. Furthermore, the WOMAC and FFQ were filled out and the SST was completed. At W4, fasting blood samples were obtained for the determination of hs-CRP and other blood markers, and the participants received the instructions and material for the urine and fecal samples, to be handed in at the W8 visit. Apart from filling out the FFQ, the W8 visit was the same as the baseline visit; in addition, participants returned what was left of their study products (i.e., cans and pills), which were later used for evaluation of participant compliance. For more information, see the parallel study with older participants that was performed in Örebro, Sweden [31].

### 4.3. Study Outcomes

#### 4.3.1. Blood Plasma, Serum, Fecal, and Urine Collection and Measurements

At W0, W4 and W8, blood samples were collected in two ethylenediaminetetraacetic acids (EDTA)-coated tubes, one serum tube, and one lithium heparin plasma tube. One plasma tube was used for blood count analysis (Sindelfingen laboratory GbR, Sindelfingen, Germany). The other one was centrifuged at 500× *g* for 7.5 min at 15 °C, followed by plasma separation, which was subsequently stored at −80 °C until FA analysis. Serum tubes were centrifuged for 15 min at 3000× *g* at 15 °C. Serum was used for quantification of gamma-glutamyl transferase (γ-GT), aspartate aminotransferase (AST), alanine transaminase (ALT), hs-CRP, plasma glucose, uric acid, triglycerides, cholesterol, high-density lipoprotein (HDL), low-density lipoprotein (LDL) and vitamin D (all measured at Laborärzte Sindelfingen GbR, Sindelfingen, Germany). The serum tube was used for the evaluation of inflammatory markers (IFN-γ, IL-4, IL-6, IL-8, IL-10, IL-12, and TNF-α) and the lithium heparin plasma for I-FAB measurement; both were stored at −80 °C and later shipped on dry ice to Orebro University, Orebro, Sweden, where the analyses took place.

The stool samples were collected before the W0 and W8 visit (max. two days before) in two tubes and stored at −20 °C in the participant’s home until delivery to the laboratory or, alternatively, when convenient the samples were transported directly to the laboratory by the study participant. Once at the laboratory, the two tubes were stored at −80 °C until the analysis commenced. One of the tubes was used to measure the gut barrier marker zonulin and the other was used for SCFA analysis. Urine for intestinal permeability measurements was collected at baseline (W0) and at the end of the intervention (W8) at home during 24-h, as described later. These samples were frozen at −80 °C and stored until further transport to Orebro University.

#### 4.3.2. Quantification of Plasma Fatty Acids

Plasma FAs were quantified according to the method of Stiefvatter et al. [72]. In short, 100 μL of plasma was supplemented with 2 μL of the internal standard solution (10,11-dichloro-undecanoic acid, DC-11:0) and 2 mL of 1% sulfuric acid in methanol (Carl Roth, Karlsruhe, Germany) for the conversion of fatty acids into fatty acid methyl esters (FAME), according to the method of Thurnhofer et al. [73]. Gas chromatography with mass spectrometry in the selected ion monitoring mode (GC/MS-SIM) was performed with the internal standards myristic acid ethyl ester according to Thurnhofer and Vetter [74]. Measurements included the unsaturated FAs listed on the following: (i) fatty acids with one double bond (monounsaturated fatty acids, MUFAs): 14:1 n-5 (myristoleic acid), 16:1, 16:1 n-7 (palmitoleic acid), 17:1, 18:1 n-9 (oleic acid), 18:1 (isomer of oleic acid), and 20:1 n-9 (gondoic acid); (ii) fatty acids with two or more double bonds (polyunsaturated fatty acids, PUFAs): 18:2 n-6 (linoleic acid, LA), 20:2 n-6 (eicosadienoic acid), 18:3 n-6 (γ-linolenic acid), 18:3 n-3 (ALA), 20:3 n-6 (dihomogammalinolenic acid), 20:4 n-6 (AA), 20:5 n-3 (EPA), 22:5 n-3 (DPA), and 22:6 n-3 (DHA).

The n-6/n-3 ratio was calculated from the summed amounts of 18:2 n-6, 20:2 n-6, 18:3 n-6, 20:3 n-6, and 20:4 n-6) divided by the summed amounts of 18:3 n-3, 20:5 n-3, 22:5 n-3, and 22:6 n-3. The AA/EPA ratio was derived from the percentage contributions of both fatty acids to the total fatty acids.

#### 4.3.3. Inflammatory Markers and Multi-Sugar Urinary Recovery Test

The measurements of the inflammatory markers IFN-γ, IL-4, IL-6, IL-8, IL-10, IL-12, and TNF-α were performed at Örebro University, Sweden, using the V-PLEX Proinflammatory Panel 1 Human Kit (MSD^®^, Rockville, MD, USA, catalog #: K15049D-1) in accordance with the manufacturer’s protocol. Two-fold dilutions of samples and controls were used; plates were incubated at 750 rpm and analyzed approximately three minutes after adding the read buffer using the MSD QuickPlex Multiplex plate reader.

Furthermore, the multi-sugar test was used for the detection of alterations in gastrointestinal permeability, as previously described [31]. In brief, the participants drank five sugars (1 g sucralose (Bulik Powders.com, Cholester, UK), 1 g erythritol (Ingredi, Baltimore, MD, USA) 1 g lactulose (Solactis, Jouy-en-jonas Cedex, France), 0.5 g rhamnose (Sigma-Aldrich/Merck, Darmstadt, Germany) and 1 g sucrose (EDEKA, Stuttgart, Germany) dissolved in water after a night of fasting. The participants remained fasting for the next five hours, and all urine expelled during this period was collected in a jar (kept cooled) for future estimation of gastroduodenal and small intestinal permeability. After these hours, the fasting ended, and all urine expelled for the next 19 h (i.e., 6–24 h after the sugar ingestion) was collected in another jar to allow for large intestinal permeability evaluation. During these 19 h, the subjects were allowed to eat and drink but were told to avoid beverages and food products that contained any of the sugars included in the multi-sugar mix. The sample preparation and determination were previously described [31] and performed in Örebro, Sweden, using high-performance liquid chromatography-mass spectrometry. The estimated sugar recovery was then used as an indicator of permeability in the following gastrointestinal segments: gastroduodenal: sucrose (0–5 h fraction); small intestine: lactulose/rhamnose (L/R) (5–19 h); large intestine: S/E (5–24 h fraction); whole gut permeability: sucralose (0–24 h fraction).

#### 4.3.4. Analysis of Intestinal Permeability Markers

The measurement of I-FABP levels was performed in Örebro, Sweden, using an enzyme-linked immunosorbent assay (I-FABP, Human, ELISA kit, HK406, Hycult Biotech, Uden, The Netherlands), following the manufacturer’s instructions. Furthermore, the fecal samples used for zonulin measurement were diluted to the recommended working concentration in stool sample tubes (K6998SAS; Immundiagnostik AG, Bensheim, Germany) using sample buffer. Zonulin was measured using a commercial enzyme-linked immunosorbent assay kit (K5600, Immundiagnostik AG, Bensheim, Germany) following the manufacturer’s instructions, as described before [75]. Fecal SCFA analysis was performed using gas chromatography (Clarus 690, Perkin-Elmer, Waltham, MA, USA) according to a previously described protocol [75].

#### 4.3.5. Sit-to-Stand Test (SST), Western Ontario and McMaster Osteoarthritis Index (WOMAC), and Food Frequency Questionnaire (FFQ)

For the evaluation of physical function, the SST was conducted as previously described [31]. Additionally, the WOMAC questionnaire was administered to the participants at W0 and W8. WOMAC registers self-reported signs of physical disability and relevant changes in health status; it was initially developed for patients with osteoarthritis, but may be used in other populations to evaluate the effects of interventions [76]. The questionnaire consists of three scales: pain, stiffness, and function. The higher the WOMAC value, the higher the pain, stiffness, and functional limitations; the maximum score is 96 (the maximum score for pain is 20, 8 for stiffness, and 68 for functional limitations), as previously described [75]. The dietary pattern was documented by a validated German FFQ [77].

#### 4.3.6. Study Products, Randomization, and Blinding

The active study product consisted of 1100 mg fish oil, 640 mg omega-3 FAs (300 mg EPA and 220 mg DHA) (dosed as 2 capsules), and 200 IU of vitamin D (VNP Active n-3 PUFA manufactured by Pfizer CH). The probiotic product contained 1.25 × 10^9^ colony-forming units (CFU) (dosed as 2 capsules, i.e., 10 billion CFUs in total) of each *Lactobacillus paracasei* (LCP-37), *Lactobacillus acidophilus* (NCFM^®^), *Bifidobacterium lactis* (Bi-07), and *Bifidobacterium lactis* (Bi-04) (Bifiform Daily Plus, supplied by Pfizer CH, manufactured by Danisco, DuPont, Wilmington, DE, USA). The control group received maltodextrin capsules for the probiotic placebo and capsules with sunflower oil for the omega-3 placebo. During the eight-week intervention, the participants had to consume four capsules daily before breakfast at the same time each day. The placebo products had the same packaging as the non-placebos and were manufactured on behalf of Pfizer Consumer Health Care (same as the active study products). To meet the compliance criteria, participants had to achieve a compliance of 85% and should have taken the supplements according to the instructions at least five days (out of seven) before the study visit, which was documented by a daily dairy.

Randomization and blinding were performed by team members not involved in the study. Thus, both participants and any study personnel who were in contact with participants were blinded. Blinding was maintained until the statistical analyses of the primary outcome parameter were completed. The randomization was generated using the Randlist software version 1.5 (datinf GmbH, Tübingen, Germany, available at randomisation.eu) and performed in blocks of four. Randomization numbers were sequentially noted on the study product containers. The study personnel handed over the lowest number available to females eligible for the study, and the highest number available to males eligible for the study. By doing so, an equal distribution of both sexes in the two arms was ensured.

### 4.4. Statistical Analyses

The statistical analysis was performed using SPSS Version 27.0 (IBM, New York, NY, USA) and for visualization, GraphPad Prism version 9.3.1 (GraphPad Software, San Diego, CA, USA) was used. Before the data analysis, a test on the normal distribution was performed (the Shapiro–Wilk test). The student *t*-test was performed for normally distributed, independent data and two samples (group comparison), and the paired *t*-test was performed for two dependent samples (within-group comparison). For non-normally distributed data and two independent samples, the Mann–Whitney U test was conducted, and the Wilcoxon test was performed in the case of dependent samples. Furthermore, the Friedman test was used (on non-normally distributed data) when more than two independent samples were compared. The treatment effect was measured by the data at the study end (W8) minus the data at the study start (W0) and expressed as delta (ΔW8−W0). All groups were tested within the study changes, comparing W0 and W8 and the treatment effect. Due to the proof-of-concept nature of this study, raw values were used for between-group comparisons before (W8) and after the treatment period at eight weeks, as well as treatment effects that were compared and tested for statistical differences. As this was an exploratory study, we also conducted within-group comparisons to generate hypotheses for future studies.

The correlation analysis from the study start and study end was calculated as Spearman’s rho. A set of planned sub-analyses was conducted, including only the individuals with obesity (BMI ≥ 30 kg/m^2^); only women with obesity; only subjects with a vitamin D level higher than 20 ng/mL at W0; and the whole study sample divided into two age categories (26–50-year-old and 51–65-year-old subjects). We also had the opportunity to include data from a parallel study of elderly subjects (n = 76) conducted in Örebro, Sweden, and registered under the same clinicaltrial.gov ID [31]. Because the interventional design was the same as in the current study, we pooled the data to increase power, reaching n = 152 for a set of Spearman’s rho correlations. As the study was an initial proof-of-concept investigation, no adjustments were made for multiple comparisons.

## 5. Conclusions

In conclusion, this proof-of-concept trial is one of the first of its kind. However, it failed to confirm the underlying hypothesis of a decrease in hs-CRP levels after eight weeks of supplementation with n-3 PUFAs, probiotics, and vitamin D in subjects with overweight and obesity. Our result may indicate that hs-CRP measurements might not be the most suitable inflammatory marker to use in this manner, as they showed substantial intraindividual variation. Potential indications of a slight anti-inflammatory effect were provided by a subtle reduction in IL-6 levels as well as improvements in the n-6/n-3 ratio and AA/EPA ratio within the treatment group. In addition, the treatment group participants showed mobility improvements, which may be a particularly valuable effect in this population. The results suggest that the dual administration of probiotics, omega-3 FAs, and vitamin D may have some modest effects on inflammation. However, on the basis of the overall lack of significant results in this study, we would recommend higher doses and longer intervention times in future studies. We are still inclined to regard these supplements as a potentially promising non-pharmacological treatment for low-grade inflammation in overweight people, but the results from this study are inconclusive and further studies are needed.

## Figures and Tables

**Table 1:** Participants’ baseline characteristics, blood biomarkers results, and the FFQ (screening).

	Placebo (n = 39)	Treatment (n = 37)	*p*-Value
Women/men (n)	30/9	30/7	
Anthropometry			
Age [years]	49 ± 10	50 ± 11	0.695
Weight [kg]	97.6 ± 12.3	100.9 ± 14	0.276
BMI [kg/m^2^]	33.8 ± 3.6	35.0 ± 2.9	0.133
n overweight	8	0	
n obese	31	37	
Blood biomarkers			
hs-CRP [mg/L]	4.1 ± 2.0	4.4 ± 2.1	0.433
γ-GT [U/L]	33 ± 24	29 ± 15	0.578
AST [U/L]	30 ± 31	26 ± 11	0.932
ALT [U/L]	28 ± 17	29 ± 14	0.445
Plasma glucose [mg/dL]	90 ± 10	95 ± 14	0.123
Insulin [µE/mL]	19.0 ± 15.7	25.2 ± 28.5	0.856
HOMA index	4.2 ± 3.5	6.2 ± 7.8	0.735
Creatinine [mg/dL]	0.83 ± 0.15	0.79 ± 0.15	0.253
Uric acid [mg/dL]	5.9 ± 1.4	5.6 ± 1.1	0.295
Cholesterol [mg/dL]	236 ± 53	231 ± 42	0.660
Triglycerides [mg/dL]	169 ± 78	148 ± 64	0.257
HDL cholesterol [mg/dL]	52 ± 12	53 ± 11	0.294
LDL cholesterol [mg/dL]	154 ± 39	150 ± 30	0.619
LDL/HDL cholesterol quotient	3.1 ± 0.9	2.9 ± 0.7	0.367
FFQ			
Energy [kcal]	1904.0 ± 867.2	1816.0 ± 896.4	0.560
Fat [g]	75.4 ± 41.6	75.3 ± 40.0	0.890
Saturated fatty acids [g]	34.6 ± 20.4	36.0 ± 20.8	0.687
Polyunsaturated fatty acids [g]	10.8 ± 5.8	9.5 ± 5.0	0.299
Short chain fatty acids [g]	1.7 ± 1.2	1.9 ± 1.2	0.641
Vitamin D [μg]	4.2 ± 3.2	3.8 ± 2.5	0.832

Values are expressed as mean ± standard deviation (SD). Abbreviations: BMI, body mass index; hs-CRP, high-sensitivity C-reactive protein; γ-GT, gamma-glutamyl transferase; AST, aspartate aminotransferase; ALT, alanine transaminase; HOMA index, Homeostasis Model Assessment for Insulin Resistance; HDL, high-density lipoprotein; LDL, low-density lipoprotein; FFQ, Food Frequency Questionnaire. Statistics: Comparison of groups revealed no difference for all parameters listed in the table (*p* > 0.05, unpaired *t*-test).

**Table 2:** Laboratory parameters before and after the intervention and fatty acid composition in plasma at weeks 0 and 8.

	Placebo			Treatment Group			Between Groups	
	W0	W8	ΔW8 − W0 Treatment Effect	W0	W8	ΔW8 − W0 Treatment Effect	*p*-Value	*p*-Value
							W8	ΔW8 − W0 Treatment Effect
Inflammatory markers								
Hs-CRP [mg/L]	4.4 ± 3.9	4.9 ± 3.6	0.51 ± 4.84	4.2 ± 2.4	5.5 * ± 3.8	1.34 ± 3.31	0.394	0.159
IL-6 [pg/mL]	0.8 ± 0.5	0.9 ± 0.6	0.08 ± 0.37	1.0 ± 0.9	0.9 * ± 0.8	−0.04 ± 0.62	0.477	0.848
TNF-α [pg/mL]	1.4 ± 0.7	1.4 ± 0.8	0.08 ± 0.33	1.3 ± 0.6	1.4 ± 0.7	0.06 ± 0.27	0.666	0.326
IFN-γ [pg/mL]	5.0 ± 2.7	6.3 ± 5.3	1.33 ± 5.44	5.3 ± 4.4	5.4 ± 4.1	0.10 ± 2.85	0.287	0.26
IL-4 [pg/mL]	0.0 ± 0.0	0.0 ± 0.0	0.00 ± 0.01	0.0 ± 0.0	0.0 ± 0.0	0.00 ± 0.02	0.477	0.427
IL-8 [pg/mL]	11.4 ± 6.3	11.5 ± 5.7	0.03 ± 2.48	19.9 ± 43.8	19.6 ± 39.2	−0.38 ± 5.46	0.856	0.856
IL-12 [pg/mL]	0.1 ± 0.1	0.1 ± 0.2	0.02 ± 0.16	0.2 ± 0.6	0.2 ± 0.6	0.02 ± 0.09	0.249	0.86
IL-10 [pg/mL]	0.3 ± 0.4	0.3 ± 0.3	0.03 ± 0.22	0.2 ± 0.1	0.2 ± 0.1	0.0 ± 0.07	0.131	0.629
Laboratory Parameters								
Weight [kg]	97.2 ± 12.1	97.4 ± 12.1	0.15 ± 2.02	101.1 ± 14.3	100.5 ± 14.2	0.17 ± 2.28	0.316	0.766
BMI [kg/m^2^]	33.7 ± 3.5	33.8 ± 3.5	0.06 ± 0.7	35.0 ± 2.8	35.1 ± 2.7	0.05 ± 0.78	0.083	0.812
Vitamin D [ng/mL]	22.4 ± 11.8	22.1 ± 10.6	−0.33 ± 5.85	24.1 ± 15.7	24.6 ± 10.9	0.51 ± 8.05	0.259	0.359
Glucose [mg/dL]	91 ± 10	93 * ± 12	1.97 ± 6.27	94 ± 12	94 ± 13	0.32 ± 6.28	0.505	0.256
Insulin [µE/mL]	14.8 ± 7.5	14.9 ± 7.3	0.04 ± 5.14	15.2 ± 8.5	14.7 ± 7.7	−0.4 ± 5.07	0.687	0.664
HOMA index	3.3 ± 1.8	3.4 ± 1.8	0.06 ± 1.35	3.5 ± 2.1	3.5 ± 1.9	0.02 ± 1.44	0.903	0.738

Abbreviations; hs-CRP, high-sensitivity C-reactive protein; IL-, Interleukin; BMI, body mass index; HOMA, Homeostasis Model Assessment for Insulin Resistance. Statistics: * differences between week 0 and W8 within the placebo and treatment groups and *p*-value at W8 as group difference. * *p* < 0.05.

## Data Availability

Not applicable.

## Supplementary Materials

The supporting information can be downloaded at: https://www.mdpi.com/article/10.3390/ijms24108567/s1.
