# Supplementary material for: Potential Modulation of Inflammation and Physical Function by Combined Probiotics, Omega-3 Supplementation and Vitamin D Supplementation in Overweight/Obese Patients with Chronic Low-Grade Inflammation: A Randomized, Placebo-Controlled Trial
Source: Int J Mol Sci. 2023 May 10;24(10):8567. doi: 10.3390/ijms24108567 (PMC10217964; doi:10.3390/ijms24108567)
Supplement: Supplementary file 1 [file ijms-24-08567-s001.zip › ijms-2369756-supplementary.pdf]

Table S1. Fatty acid changes in plasma

	**Placebo**		**Treatment group**		**Between groups**		
	W0	W8	W0	W8	p-value W0	p-value W8	p-value Δ W8- W0 Treatment effect
			Fatty acids [mg/100µl]				
14:1	0.20 ± 0.12	0.24 ± 0.20	0.25 ± 0.22	0.45 ± 0.34***	0.743	0.002	0.002
16:1	1.26 ± 0.71	1.44 ± 0.68*	1.45 ± 0.65	1.81 ± 0.62***	0.120	0.005	0.005
16:1 n-7	5.45 ± 3.14	5.91 ± 3.08	5.79 ± 2.00	7.36 ± 2.88***	0.159	0.009	0.009
17:1	0.37 ± 0.28	0.41 ± 0.31	0.41 ± 0.22	0.58 ± 0.23***	0.078	< 0.001	< 0.001
18:1 n-9 c	41.49 ± 15.46	44.24 ± 14.19	44.96 ± 17.83	46.45 ± 20.70	0.147	0.588	0.588
18:1	3.56 ± 1.78	3.79 ± 1.74	4.12 ± 1.67	5.16 ± 1.53**	0.150	< 0.001	< 0.001
20:1	0.32 ± 0.22	0.42 ± 0.42	0.36 ± 0.24	0.56 ± 0.43	0.496	0.104	0.104
18:2 n-6 (LA)	53.89 ± 20.00	60.35 ± 18.87*	60.35 ± 22.01	70.58 ± 18.97***	0.159	0.022	0.022
20:2 n-6	0.39 ± 0.16	0.42 ± 0.15	0.39 ± 0.13	0.49 ± 0.24**	0.666	0.260	0.260
18:3 n-6	1.07 ± 0.62	1.20 ± 0.63	1.40 ± 0.79	1.51 ± 0.78	0.055	0.067	0.067
18:3 n-3 (ALA)	1.26 ± 0.82	1.36 ± 0.94	1.56 ± 1.27	1.88 ± 1.25*	0.369	0.018	0.018
20:3 n-6	3.91 ± 2.16	4.50 ± 1.98*	4.56 ± 1.91	5.35 ± 1.82**	0.036	0.035	0.035
20:4 n-6 (AA)	15.25 ± 7.34	17.93 ± 7.12**	19.16 ± 6.33	20.85 ± 6.58*	0.003	0.020	0.020
20:5 n-3 (EPA)	1.70 ± 1.16	2.50 ± 4.04*	2.68 ± 1.80	5.48 ± 2.12***	0.002	< 0.001	< 0.001
22:5 n-3 (DPA)	1.85 ± 1.30	2.60 ± 1.75**	2.44 ± 1.52	3.62 ± 1.72***	0.085	0.004	0.004
22:6 n-3 (DHA)	6.46 ± 2.91	8.65 ± 6.34**	9.64 ± 4.36	12.46 ± 3.39**	< 0.001	< 0.001	< 0.001
PUFA	0.60 ± 0.41	0.73 ± 0.39	0.70 ± 0.39	0.62 ± 0.40	0.091	0.251	0.251
n-3 FA	11.27 ± 5.01	15.11 ± 12.09**	16.32 ± 6.79	23.44 ± 6.45***	< 0.001	< 0.001	< 0.001
n-6 FA	74.51 ± 29.09	84.39 ± 27.04*	85.86 ± 28.73	98.79 ± 26.10**	0.078	0.012	0.012

Means of fatty acid concentrations in plasma in mg/100 µL at study time points W0 and W8 in the treatment group and the placebo group are shown, as well as the p-value of W8 as group differences. Abbreviations: AA, arachidonic acid; ALA, α-linolenic acid; DHA, docosahexaenoic acid; DPA, docosapentaenoic acid; EPA, eicosapentaenoic acid; LA, linoleic acid; n, omega; W, week. Statistics: * differences between week 0 and W8 within the placebo group and the treatment and p-value at W8 as group difference. *$p < 0.05$; **$p < 0.01$; and ***$p < 0.001$

Table S2. Physical function tests, SCFAs and gut barrier markers

	Placebo			Treatment			Between groups	
	W0	W8	Δ W8-W0 Treatment effect	W0	W8	Δ W8-W0 Treatment effect	W8	Δ W8-W0 Treatment effect
Physical function tests								
5 STS [s]	10.9 ± 3.2	10.1* ± 3.3	-0.75 ± 1.78	11 ± 3.1	10.3* ± 2.9	-0.67 ± 2.38	0.503	0.823
WOMAC score	14.1 ± 14.5	13.3 ± 16.7	-0.8 ± 6.8	19.1 ± 17.7	15.8* ± 17.7	-3.4 ± 7.1	0.346	0.095
SCFAs [µmol/g wet mass]								
Acetic acid	61.7 ± 26.5	58.5 ± 23.2	-3.15 ± 16.05	62.5 ± 25.9	62.1 ± 21.9	-0.34 ± 17.27	0.350	0.480
Propionic acid	16.6 ± 7.6	15.4 ± 6.4	-1.13 ± 5.56	19.3 ± 8.7	17.1* ± 9.6	-2.20 ± 7.92	0.381	0.146
Iso-butyric acid	1.7 ± 0.8	1.7 ± 0.8	0.03 ± 1.04	1.6 ± 0.7	1.5 ± 0.5	-0.13 ± 0.69	0.259	0.442
Butyric acid	13.6 ± 6.9	12.1 ± 6.1	-1.52 ± 6.24	14.2 ± 7.3	14.2 ± 8.6	0.01 ± 8.04	0.362	0.371
Iso-valeric acid	2.1 ± 1.2	2.3 ± 1.1	0.11 ± 1.52	2.0 ± 0.9	1.8 ± 0.8	-0.14 ± 0.99	0.102	0.407
Valeric acid	1.9 ± 0.9	1.8 ± 0.9	-0.09 ± 1.10	1.8 ± 0.8	1.9 ± 1.0	0.02 ± 1.02	0.888	0.682
Iso-caproic acid	0.2 ± 0.2	0.2 ± 0.2	0.00 ± 0.18	0.2 ± 0.2	0.2 ± 0.2	0.02 ± 0.17	0.791	0.662
Hexanoic acid	0.5 ± 0.5	0.5 ± 0.5	-0.05 ± 0.32	0.4 ± 0.5	0.4 ± 0.5	-0.02 ± 0.45	0.636	0.440
Heptanoic acid	0.1 ± 0.1	0.1 ± 0.1	-0.01 ± 0.08	0.1 ± 0.1	0.1 ± 0.1	0.00 ± 0.08	0.534	0.407
Multi-sugar urinary recovery test								
0-5 hrs								
L/R ratio	0.176 ± 0.642	0.059 ± 0.043	-0.117 ± 0.606	0.066 ± 0.052	0.074 ± 0.059	0.008 ± 0.051	0.238	0.358
sucrose [µg/mL]	29.37 ± 44.85	31.78 ± 54.70	2.41 ± 60.09	45.17 ± 153.94	50.39 ± 110.27	5.44 ± 100.79	0.718	0.439
5-24 hrs								
S/E ratio	0.025 ± 0.010	0.031 ± 0.018	0.005 ± 0.018	0.031 ± 0.033	0.027 ± 0.010	-0.004 ± 0.030	0.541	0.541
Gut barrier markers								
I-FABP [pg/ml]	482 ± 354	500 ± 313	17.45 ± 230.90	574 ± 348	563 ± 317	4.01 ± 392.50	0.251	0.326
Zonulin	191.9 ± 137.8	185.9 ± 115.6	-6.014 ± 145,8	184.3 ± 149.3	165.6 ± 111.6	-18.69 ± 166.1	0.329	0.798

Values are given as mean ± SD. Statistic:* Shows significant differences between W0 and W8 within each group. Abbreviations: IFN-γ; interferon gamma; IL-, Interleukin-; TNF-α, tumor necrosis factor-α; W, week; L/R, lactulose/rhamnose; S/E, sucralose/erythritol. Statistics: no difference within groups and between groups.
